# Extreme weather events and death based on temperature and CO_2_ emission – A global retrospective study in 77 low-, middle- and high-income countries from 1999 to 2018

**DOI:** 10.1016/j.pmedr.2022.101846

**Published:** 2022-05-31

**Authors:** Maral Amirkhani, Shidrokh Ghaemimood, Johan von Schreeb, Ziad El-Khatib, Sanni Yaya

**Affiliations:** a Public Health Graduate Studies, Bahá’i Institute for Higher Education (BIHE), Iran; b Department of Global Public Health, Karolinska Institutet, Stockholm, Sweden; c Medical University of Vienna, Vienna, Austria; d World Health Programme, Université du Québec en Abitibi-Témiscamingue (UQAT), Québec, Canada; e School of International Development and Global Studies, University of Ottawa, Ottawa, Canada; f The George Institute for Global Health, Imperial College London, London, UK

**Keywords:** GDP, Heat wave, Cold wave, Severe winter condition, CO_2_ emission, Temperature

## Abstract

Due to rising temperatures and CO_2_ emissions, climate change has become one of the most important global issues. We described the relationship between extreme weather-related events and death, globally, from 1999 through 2018. We used data from the emergency events database of the Université Catholique de Louvain. We also categorized the countries’ income according to the World Bank GDP and we used the CO_2_ emission levels data from the Carbon Dioxide Information Analysis Center to link the GDP and CO_2_ emissions to years of extreme weather conditions in each country. We conducted descriptive and Poisson Regression analysis to analyze the data. A total of 77 countries reported 425 extreme weather-related events from1999 through 2018. Mortality related events were highest in middle-income countries due to severe winter conditions (N = 2,020) and cold-waves (N = 70,972). The total number of recorded deaths due to heat waves was highest in high-income countries (N = 84,344). Furthermore, the number of deaths in high-income countries, compared to low-income countries, was five-fold higher (IRR 5.18; 95%CI 4.58; 5.85, p < 0.001). The mortality rate in heat season was almost seven-fold higher than that in cold/severe winter (IRR 33.43; 95%CI 32.85; 34.02, p < 0.001). The number of deaths increased significantly with the repetition of extreme events (IRR 6.82; 95%CI 6.68; 6.96, p < 0.001). We found the number of deaths increased in high-income countries, and this was associated with an increase in the number of times extreme events occurred per year and with heat wave.

## Highlights

• Summary of reported number of extreme weather events/associated deaths globally.
• Data from a humanitarian disaster database (77 countries, 425 events, 1999–2018).
• Number of deaths increased significantly with the repetition of extreme events.
• Mortality rate in heat season was 7 fold higher than that in cold/severe winter.
• Consistent reporting measures, is needed, from severe weather-related events.

## 1 Introduction

The World Health Organization (WHO) estimates that, between 2030 and 2050, there will be approximately 250,000 additional deaths per year due to climate change (World Health Organization (WHO), 2021). A study from 1995 to 2015 found that 606,000 persons died during this period due to weather-related events (The United Nations Office for Disaster Risk Reduction (UNISDR), 2015). Different types of extreme weather-related events increase in frequency and severity due to climate change, including drought (Thomas et al., 2013), severe winter conditions (Sternberg, 2010), heat waves, cold waves, and floods (Curtis et al., 2017).

Increasing greenhouse gases affects temperature, which can contribute to extreme weather-related events (Thomas et al., 2013). Most studies that have investigated the relationship between extreme weather-related events and mortality have been limited to a specific geographical area, such as European regions including north Finland, south Finland, Baden-Württemburg, the Netherlands, London, and north Italy (The Eurowinter Group, 1997), or Asia and the Pacific (Thomas et al., 2013). Additionally, prior investigations have been limited to specific events like cold waves or heat waves, specific age groups like older people (Klenk et al., 2010, Marmor, 1978), or events during limited periods of time, such as evaluating four heatwaves that happened between 1972 and 1973 (Marmor, 1978). According to our knowledge, no study has assessed the relationship between death and extreme weather-related events over an extended period of time and across the globe. To address this knowledge gap, we examined the relationship between death and extreme weather-related events from 1999 through 2018. The findings of this study can provide insight for national and global planning to reduce mortality from weather-related events, as well as for environmental planning.

## 2 Methods

### 2.1 Data sources and inclusion criteria

#### 2.1.1 Emergency events data sources

Temperature data and number of deaths for all international events of heat wave, cold wave, and severe winter condition from 1999 to 2018 were obtained from the Emergency Events Database (EM-DAT) of the Université Catholique de Louvain, located in Brussels, Belgium (Guha-Sapir et al., 2019).

The EM-DAT was launched in 1988 as a humanitarian database, and it has collected data on > 21,000 disasters that have occurred globally since 1900. The classification of disasters, in EM-DAT, is based on the Integrated Research on Disaster Risk Peril Classification and Hazard Glossary (Integrated Research on Disaster Risk (IRDR), 2014).

The EM-DAT data sources include United Nations agencies, governments, the International Federation of Red Cross and Red Crescent Societies, other non-governmental organizations, insurance companies, research institutions, and press agencies (Guha-Sapir et al., 2019).

*Inclusion criteria*: One or more of the following criteria must be fulfilled for a weather event to be considered a disaster event in the EM-DAT: (1) ≥ 10 people died, (2) ≥ 100 people were affected, (3) the declaration of a state of emergency or (4) a call for international assistance. Each of the severe weather events reported in this study was qualified as a disaster event. A detailed description of the EM-DAT structure, classification, variable descriptions, and definitions is available under EM-DAT: https://www.emdatbe/guidelines.

#### 2.1.2 Country income and CO_2_ classification

The countries’ income category was determined based on the World Bank Gross Domestic Product (GDP) (World Bank, 2020) information. In addition, the CO_2_ emission levels data, which was measured as CO_2_ emissions (Kt) from the burning of fossil fuels, was downloaded from the Carbon Dioxide Information Analysis Center (Carbon Dioxide Information Analysis Center, 2017). We used this information to link the GDP and CO_2_ emission data to years of extreme weather conditions in each country. (See Appendix A for the list of the included countries).

#### 2.1.3 Definitions

The definitions used by EM-DAT were as follow:

**Extreme temperature**: a general term for temperature variations above (extreme heat) or below (extreme cold) normal conditions.

**Heat wave:** a period of abnormally hot and/or unusually humid weather.

**Cold wave:** a period of abnormally cold weather. Both weather extremes typically last two or more days. Severe winter conditions include snow, ice, frost, and freeze (Brennenstuhl et al., 2021). It should be noted that the exact temperature criteria for what constitutes a heat or cold wave varies by location (Integrated Research on Disaster Risk (IRDR), 2014). Additionally, the descriptions and definitions are available under EM-DAT page: https://www.emdat.be/classification.

### 2.2 Statistical analysis

First, we conducted descriptive analysis—which included the mean, median, minimum, maximum, and inter quartile range—to determine the number of deaths from each weather-related event, as well as temperature and CO_2_ emission, among each GDP category during the period of 1999 through 2018. Then, we used Poisson regression models to calculate incidence rate ratios (IRR) for the events of deaths due to extreme weather conditions (heat waves, cold waves, and severe winter weather).

Ethical approval was not required, as this study did not include human subjects.

## 3 Results

Our descriptive analysis showed that the total number of extreme weather-related events from 1999 through 2018 was 422 in 77 countries. The distribution of these events was 15 (4%), 227 (53%), and 183 (43%) for low-, middle-, and high-income countries, respectively (Table1).

**Table 1:** Total number of countries, deaths, and extreme weather-related events during 1990 through 2018.

	**Total Number**	**Low-income Countries**	**Middle-income** **Countries**	**High-income** **Counties**
**Number of countries**	77	4	39	34
**Number of Extreme weather-related events**	**422**	**15**	**227**	**183**
Severe winter conditions	65 (15.4%)	2 (13.3%)	38 (16.7%)	25 (13.7%)
Cold waves	220 (52.1%)	12 (80%)	126(55.5%)	82 (44.8%)
Heat waves	137 (32.5%)	1 (6.7%)	60 (26.4%)	76 (41.5%)
**Total number of deaths**	**172,039**	**2,201**	**82,334**	**87,504**
Severe winter conditions	3,794 (2.2%)	1,362 (61.9%)	2,020 (2.4%)	412 (0.5%)
Cold waves	74,559 (43.3%)	839 (38.1%)	70,972 (86.2%)	2,748 (3.1%)
Heat waves	93,686 (54.5%)	0 (0%)	9,342 (11.4%)	84,344 (96.4%)

The total number of recorded deaths in extreme weather-related events was 172,039. The number of deaths that occurred were 2,201 (1%), 82,334 (48%) and 87,504 (51%) in the respective order from low to high-income countries level. The number of deaths due to severe winter conditions was highest in middle-income countries (N = 2,020; 53%) followed by high-income (412; 11%) and low-income countries (1,362; 36%) respectively. The total number of recorded deaths due to cold waves was 70,972 (95%) in middle-income countries, 2,748 (4%) in high-income countries, and 839 (1%) in low-income countries. The number of deaths due to heat waves was only reported in high-income (n = 84,344 (90%) and middle-income countries (n = 9,342 (10%)) (Table 1).

The average temperature due to severe winter conditions was lowest in high-income countries (−27.0 °C), followed by middle-income (−24.5 °C) and low-income (−18.0 °C) countries. The average temperature due to cold waves was lowest in high-income countries (–23.2 °C) (Table 2). The average CO_2_ emission was 0.2Kt, 3.9Kt and 8.7Kt in low-, middle- and high-income countries, respectively (Table 2). The number of deaths in high-income countries, compared to low-income countries, was five-fold higher (IRR 5.18; 95%CI 4.58; 5.85, p < 0.001). The mortality rate during heat waves was almost seven-fold higher than deaths caused by cold waves/severe winter weather (IRR 33.43; 95%CI 32.85; 34.02, p < 0.001) (Table 3). The number of deaths increased significantly with the repetition of extreme events (IRR 6.82; 95%CI 6.68; 6.96, p < 0.001) (Table 3).

**Table 2:** Mean and Median of the temperature (^o^C) and CO_2_ emission (Kt) of the extreme weather-related events during 1990 through 2018.

	**Total Number**		**Low-income Countries**		**Middle-income Countries**		**High-income Counties**	
	**Mean (min**–**max)**	**Median (IQR)**	**Mean (min**–**max)**	**Median (IQR)**	**Mean (min**–**max)**	**Median (IQR)**	**Mean (min**–**max)**	**Median (IQR)**
**Temperature (^o^C)**								
**Total**	−0.2 °C (−57; 60)	−13 °C (−25; 38)	−13.6 °C (−25; 37.8)	−18 °C (−20; −15)	−0.9 °C (−57 °C; 60 °C)	−10 °C (−25; 36)	1.8 °C (−48; 52)	−20 °C (−25; 39)
**Severe winter conditions**	−25.5 °C (−55; 8)	−25 °C (−35; −20°)	−18 °C (−18; −18)	−18 °C (--)	−24.5 °C (−55 °C; 8 °C)	−25 °C (–32; −20)	−27.8 °C (−40; 0)	−31 °C (−35; −20.75)
**Cold waves**	−18.5 °C (−57; 8.6)	−20 °C (−25; −10)	−17.1 °C (−25; 0)	−20 °C (−20; −15)	−15.5 °C (−57 °C; 8.6 °C)	−15 °C (−25; 5)	–23.2 °C (−48; −1.3)	−25 °C (−26; −20)
**Heat waves**	41.9 °C (32; 60)	40 °C (39; 45.6)	37.8 °C (--)	37.8 °C (--)	44 °C (32 °C; 60 °C)	44.5 °C (40; 47)	40.3 °C (32; 52)	42 °C (37.3; 43)
**CO_2_ emission (Kt)**								
**Total**	5.6Kt (0.04;22.1)	4.7 Kt (1.7; 7.8)	0.2 Kt (0.04; 0.4)	0.2 Kt (0.12; 0.22)	3.9 Kt (0.2; 15.3)	2.8 Kt (1.03; 4.99)	8.7 Kt (1.6; 22.1)	7.8 Kt (5.6; 9.9)
**Severe winter conditions**	5.5 Kt (0.15; 18.5)	5.16 Kt (1.96; 7.13)	0.25 Kt (0.15; 0.35)	0.25 Kt (0.2; 0.3)	4.11 Kt (0.3; 15.3)	4.05 Kt (1.6; 6.5)	8 Kt (1.8; 18.5)	7.3 Kt (5.6; 9)
**Cold waves**	4.8 Kt (0.04; 19.7)	4.17 Kt (1.6; 7.4)	0.17 Kt (0.04; 0.28)	0.19 (0.11; 0.21)	3.6 Kt (0.15; 13.1)	3.3 Kt (0.98; 4.9)	7.5 Kt (1.6; 19.7)	7.31 Kt (4.7; 9.07)
**Heat waves**	6.9 Kt (0.26; 22.1)	5.75 Kt (2.34; 9.44)	–	–	2.7 Kt (0.26; 11)	1.73 Kt (0.9; 4.2)	10.3 Kt (3.9; 22.1)	8.8 Kt (6.7; 11.5)

**Table 3:** Incidence Rate Ratio (95% Confidence Interval) for death events during 1990 through 2018.

**Characteristics**	IRR*	**(95% CI)**	**p-value**
**Country income**			
Low-income	1		
Middle-income	1.09	(0.96; 1.23)	0.19
High-income	5.18	(4.58; 5.85)	<0.001
**Number of times extreme event occurred per year**	33.43	(32.85; 34.02)	<0.001
**Type of disaster**			
Cold/severe winter	1		
Heat	6.82	(6.68; 6.96)	<0.001

* Adjusted for year, CO_2_ emission, population size and Gini.

## 4 Discussion

In this study, we reported the number of deaths due to three types of extreme weather-related events (heat wave, cold wave, severe winter conditions) within low-, medium-, and high-income countries. We found that the number of deaths due to heat waves was the highest in high-income countries (84,344; 90%), followed by middle-income (9,342; 10%), whereas low-income countries had no reported deaths. It seems there are risk factors in high income countries compared to LMIC that increase the risk of deaths from heat waves. The age of the population (Centers for Disease Control and Prevention, 1995), lack of acclimatization, low fitness, and obesity are the main risk factors for heat-related illness. Other important heat-related risk factors are dehydration due to lack of liquid uptake, intestinal problems, use of diuretics, and alcohol abuse (World Health Organization (WHO) – Europe, 2004). As WHO reported, there is a significant difference in life expectancy between high- and LMIC. As older people are more vulnerable to the risk of heat wave death due to disturbance of homeostasis, weaker cardiovascular system, underlying diseases, use of certain medication, cognitive impairment, living alone and decreased fitness (WHO, Health and Global Environmental Change Series No. 2, 2004), these factors may explain the increase in death due to heat waves in high-income compared with low-income countries.

Short-term heat acclimatization usually takes 3–12 days, but complete (long-term) acclimatization to an unfamiliar thermal environment may take several years (Ebi and Meehl, 2007). This means that people living in warmer areas might be more adaptable to rising temperatures in the long term, while sudden increases in temperature changes can be fatal for people living in the cold area. Numerous factors, including proper policymaking and access to wealth resources, can help control deaths due to rising temperatures. However, studies have shown that the number of deaths and temperature changes can vary with latitude. For example, the results of a study in 11 cities of the Eastern United States showed that the latitude of the city is associated with the temperature-mortality relation. Colder temperatures in more southern cities and warmer temperatures in more-northern cities have a greater effect on the risk of mortality (Curriero et al., 2002). The results of a Euro-winter study also showed that with decreasing temperature in areas with warmer winters, the mortality rate increased more (The Eurowinter Group, 1997).

The lack of reported deaths from heat waves in low-income countries may be due to lack of an efficient system for recording mortality information. Other studies have emphasized the importance of consistent reporting measures, especially in LMIC, especially since the mortality risk due to climate change disproportionately falls on low-income countries (Carleton et al., 2020). Heat can be even more threatening than cancer and heart disease today and could be responsible for an additional 200 or more deaths per 100,000 people by the end of the century, in hot and low-income countries (Climate Impact Lab, 2020). One risk factor that can affect the heat wave related mortality in poorer countries is the lack of access to the healthcare facilities (Centers for Disease Control and Prevention, 1995).

There is a growing link between climate change and non-communicable diseases (NCD). For example, extreme weather conditions due to climate change can be a growing threat for cancer patients to access their treatment facilities (Nogueira et al., 2020, Ryan et al., 2015). Internationally, policymakers have considered climate change to be ranked as a top threatening priority, even above non-communicable diseases (Colagiuri et al., 2015).

We also found that the average CO_2_ emissions due to heat-wave, cold-wave, and severe winter conditions was higher in high-income countries. Also, we found a significant correlation between the total number of deaths and CO_2_ emissions in cold wave events among middle-income countries. One factor which can cause more CO_2_ emission is urbanization.

One study assessing the effects of urbanization on CO_2_ emissions investigated how four different aspects of urbanization affect CO_2_ emissions. The results showed that economic urbanization and land urbanization each have a positive impact on CO_2_ emissions. This impact is due to the changes in the non-built to built area and accumulation of wealth, while population and social urbanization have a negative impact on CO_2_ emissions (Wang et al., 2018). In addition to the urbanization of countries, the national culture also has a significant impact on the relationship between CO_2_ emissions and economic growth (Disli et al., 2016). As a result, it is not far-fetched that in our study, CO_2_ emissions in developed countries were higher than in low-income countries. Also, the relationship between number of deaths from cold waves and CO_2_ emission among middle-income countries was significant.

## 5 Study limitations

Although our study presented novel results, we should list a few study limitations. The data depends on the countries’ reporting system and how they were collected. Therefore, we think there might have been a variation in the data collection methods between low-, middle-, and high-income countries. Additionally, the duration of each extreme weather-related event can affect the death rate. Unfortunately, we could not assess the duration of each event as it was not reported. Additionally, a major confounder in this study was the lack of information regarding how long an extreme weather-related event will take. For example, in this study, we found that heat wave deaths were higher in high-income countries. As mortality is related to the duration of the heat wave (Tan et al., 2007), elongation of the heat wave may play a greater role in human death compared to other factors like temperature.

## 6 Conclusion

The results of this study show that the majority of cold wave related deaths occurred in middle-income countries followed by high-income countries. We also found that more deaths were likely to occur during heat waves than cold waves or severe winter weather, in particularly in high-income countries. Finally, increased CO_2_ emissions can result in an increase in the number of deaths during severe weather events.

## Funding

No funding was received for this study.

### CRediT authorship contribution statement

**Maral Amirkhani:** Conceptualization, Writing – original draft, Data curation, Writing – review & editing. **Shidrokh Ghaemimood:** Conceptualization, Supervision, Writing – review & editing. **Johan von Schreeb:** Writing – review & editing. **Ziad El-Khatib:** Conceptualization, Supervision, Writing – review & editing. **Sanni Yaya:** Conceptualization, Writing – review & editing.

## Declaration of Competing Interest

The authors declare that they have no known competing financial interests or personal relationships that could have appeared to influence the work reported in this paper.
