# Supplementary material for: Extreme weather events and death based on temperature and CO2 emission – A global retrospective study in 77 low-, middle- and high-income countries from 1999 to 2018
Source: Prev Med Rep. 2022 May 31;28:101846. doi: 10.1016/j.pmedr.2022.101846 (PMC9163583; doi:10.1016/j.pmedr.2022.101846)
Supplement: Supplementary data 1 [file mmc1.docx]

**Appendix A. List of countries, distributed by low-, middle- and high-income categories**

| **GDP income category** | **Country name** |
| --- | --- |
| Low-income (n=4) | Afghanistan |
|  | Liberia |
|  | Nepal |
|  | North Korea |
| Middle-income (n=39) | Albania |
|  | Algeria |
|  | Argentine |
|  | Armenia |
|  | Azerbaijan |
|  | Bangladesh |
|  | Belarus |
|  | Belize |
|  | Bolivia |
|  | Bosnia and Herzegovina |
|  | Brazil |
|  | Bulgaria |
|  | China |
|  | Egypt |
|  | El Salvador |
|  | Guatemala |
|  | India |
|  | Jordan |
|  | Kazakhstan |
|  | Kyrgyzstan |
|  | Macedonia |
|  | Mexico |
|  | Moldova |
|  | Mongolia |
|  | Montenegro |
|  | Morocco |
|  | Nigeria |
|  | Pakistan |
|  | Paraguay |
|  | Peru |
|  | Romania |
|  | Russia |
|  | Serbia |
|  | South Africa |
|  | Sudan |
|  | Tajikistan |
|  | Thailand |
|  | Turkey |
|  | Ukraine |
| High-income (n=34) | Austria |
|  | Australia |
|  | Belgium |
|  | Canada |
|  | Canary Island |
|  | Chile |
|  | Croatia |
|  | Cyprus |
|  | Czech republic |
|  | Estonia |
|  | France |
|  | Germany |
|  | Greece |
|  | Hungary |
|  | Israel |
|  | Italy |
|  | Japan |
|  | Latvia |
|  | Lithuania |
|  | Luxembourg |
|  | Netherlands |
|  | New Zealand |
|  | Poland |
|  | Portugal |
|  | Slovakia |
|  | Slovenia |
|  | South Korea |
|  | Spain |
|  | Sweden |
|  | Switzerland |
|  | Taiwan |
|  | United Kingdom |
|  | United states of America |
|  | Uruguay |
